# A Semantic-Associated Factor Graph Model for LiDAR-Assisted Indoor Multipath Localization

**DOI:** 10.3390/s26010346

**Published:** 2026-01-05

**Authors:** Bingxun Liu, Ke Han, Zhongliang Deng, Gan Guo

**Affiliations:** 1 School of Electronic Engineering, Beijing University of Posts and Telecommunications, Beijing 100876, China; hanke@bupt.edu.cn (K.H.); dengzl@bupt.edu.cn (Z.D.); 2 Key Laboratory of Mobile Application Innovation and Governance Technology, Ministry of Industry and Information Technology, Beijing 100191, China; guogan@caict.ac.cn

**Keywords:** LiDAR, multipath, factor graph optimization, indoor position, virtual anchor

## Abstract

In indoor environments where Global Navigation Satellite System (GNSS) signals are entirely blocked, wireless signals such as 5G and Ultra-Wideband (UWB) have become primary means for high-precision positioning. However, complex indoor structures lead to significant multipath effects, which severely constrain the improvement of positioning accuracy. Existing indoor positioning methods rarely link environmental semantic information (e.g., wall, column) to multipath error estimation, leading to inaccurate multipath correction—especially in complex scenes with multiple reflective objects. To address this issue, this paper proposes a LiDAR-assisted multipath estimation and positioning method. This method constructs a tightly coupled perception-positioning framework: first, a semantic-feature-based neural network for reflective surface detection is designed to accurately extract the geometric parameters of potential reflectors from LiDAR point clouds; subsequently, a unified factor graph model is established to multidimensionally associate and jointly infer terminal states, virtual anchor (VA) states, wireless signal measurements, and LiDAR-perceived reflector information, enabling dynamic discrimination and utilization of both line-of-sight (LOS) and non-line-of-sight (NLOS) paths. Experimental results demonstrate that the root mean square error (RMSE) of the proposed method is improved by 32.1% compared to traditional multipath compensation approaches. This research provides an effective solution for high-precision and robust positioning in complex indoor environments.

## 1. Introduction

**Figure 1:**
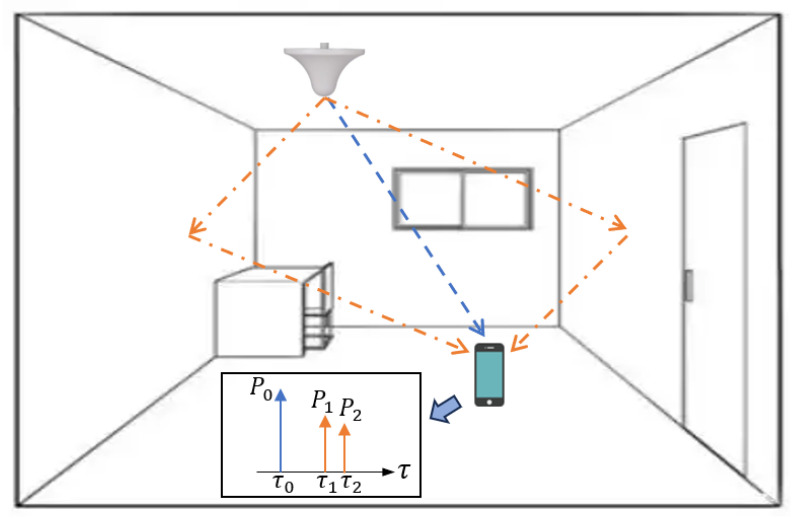
Multipath effects in indoor environments.

The integration of multi-sensor technologies such as LiDAR and cameras to assist navigation has become a pivotal trend in the future development of mobile network communication and positioning. In outdoor environments, the Global Navigation Satellite System (GNSS) remains the primary method for providing absolute positioning information. However, in challenging scenarios like urban canyons, GNSS signals are susceptible to obstruction by buildings, leading to Non-Line-of-Sight (NLOS) propagation and a significant degradation in positioning accuracy [1]. Consequently, substantial research efforts are dedicated to the identification and mitigation of NLOS errors. Additionally, multipath effects further exacerbate ranging inaccuracies [2], as illustrated in Figure 1. In GNSS-denied indoor environments such as parking garages and shopping malls, technologies capable of high-precision ranging, including 5G [3] and Ultra-Wideband (UWB) [4], are widely employed for indoor positioning. Nonetheless, the complex layout of indoor settings intensifies multipath effects, often involving a high density of short-delay multipath components [5], which renders traditional multipath suppression methods designed for outdoor scenarios less effective. Therefore, investigating efficient multipath estimation and high-accuracy positioning methods is essential for enhancing localization performance in complex indoor environments.

Addressing the aforementioned challenges, existing research primarily focuses on two aspects. The first involves identifying and modeling propagation paths based on the characteristics of received signals. Statistical analysis of channel characteristics can be used to model multipath components [6]. Multipath estimating delay lock loops can also analyze multipath component errors [7]. The second approach utilizes advanced signal processing or data fusion algorithms to mitigate multipath effects. In terms of signal processing, filtering algorithms such as Kalman Filter (KF) and Particle Filter (PF) are often employed to smooth signal measurements [8]. For fusion-based positioning, Inertial Measurement Units (IMU), LiDAR, and Visual-Inertial Odometry (VIO) are common fusion sources used to reduce positioning errors from a single source [9]. In the field of environmental perception, LiDAR has gained significant attention due to its ability to directly acquire high-precision 3D environmental point cloud data. Semantic information extraction from LiDAR point clouds (such as reflective surfaces like walls, doors, and windows) provides geometric constraints for understanding the signal propagation environment [10]. Regarding multipath processing, tracking methods based on filtering or factor graph optimization are used to estimate the state of multipath [11]. However, effectively correlating perceptual information with signal observations and, on this basis, achieving joint optimization of multipath components and positioning states remains a research challenge.

This paper proposes a semantic association-based multipath estimation and localization method. Its core innovation lies in constructing a tightly coupled perception-localization framework, which aims to enhance positioning accuracy in indoor environments through multi-source information fusion. The method first designs a semantic feature-based neural network for reflective surface detection. This network can accurately extract geometric parameters (e.g., center position, normal vector) of potential reflectors from LiDAR point clouds. Through this extraction, a potential association between semantic perception and multipath propagation is established. Subsequently, a unified factor graph model is established to achieve multi-dimensional association and joint inference estimation of terminal states, virtual anchor states, wireless signal measurements, and LiDAR-perceived reflective surface information. This model effectively handles the complex data association between terminal motion, signal multipath components, historical virtual anchor states, and LiDAR perception synchronously, enabling dynamic discrimination and utilization of line-of-sight, non-line-of-sight, and multipath signals. Validation through experiments in real indoor scenarios demonstrates significant improvements in both positioning accuracy and robustness.

The remainder of this paper is organized as follows: Section 2 reviews related work. Section 3 elaborates on the LiDAR-based reflective surface detection method. Section 4 details the factor graph-based multipath estimation and localization model. Section 5 presents the experimental setup and results analysis. Finally, the Section 6 are provided.

## 2. Related Work

In this section, we categorize the work related to this paper into three parts: LiDAR-based semantic perception methods, multipath processing approaches for localization and navigation, and multipath estimation techniques based on object tracking. We briefly introduce the principles of these methods.

### 2.1. LiDAR-Based Semantic Perception

Currently, LiDAR has become an essential sensor in autonomous driving, robotics, and geographic information mapping. LiDAR captures three-dimensional (3D) point cloud data frames and is widely used in semantic understanding tasks such as semantic segmentation, scene classification, and object recognition. LiDAR point cloud semantic tasks are primarily divided into traditional methods and deep learning-based methods. Traditional methods typically involve manually extracting geometric features and outputting results through classifiers such as Support Vector Machines (SVMs) [12] or Random Forests (RFs) [13]. Deep learning-based methods employ neural networks to learn the feature extraction process. These neural networks output semantic labels in an end-to-end manner. Depending on the data input format, they can be further categorized into point-based methods, image-based methods, voxel-based methods, and graph-based methods. Point-based methods directly take raw point clouds as input, making them suitable for any unstructured point cloud. The main challenge in raw point cloud processing lies in extracting local contextual features from cluttered point clouds. PointNet [14] is a pioneer in point-based deep networks for unstructured point cloud processing. It employs shared multilayer perceptrons (MLPs) to extract per-point features and aggregates global features through a symmetric max-pooling operation. PointCNN [15] constructs ordered feature sequences by sorting the K nearest points to a central point, enabling convolutional neural network (CNN) operations. The SqueezeSeg [16] series generates range images by projecting a frame of 3D LiDAR data onto a spherical surface, followed by building image-based semantic segmentation models. Voxel-based methods [17] first convert unordered 3D LiDAR data into ordered voxel data, then predict semantic labels using 3D CNNs. Graph-based methods [18] construct graphs from 3D LiDAR data, encoding vertex features and employing graph convolutions to extract contextual information.

### 2.2. Multipath Processing for Localization and Navigation

In multipath scenarios, the received signal typically comprises a mixture of reflected echoes and scattered echoes. Influenced by factors such as path delay, signal strength, and receiver loop design, the ranging error introduced in the receiver can range from several meters to several tens of meters [19]. The Code-Minus-Carrier (CMC) observable is commonly used to study multipath characteristics and mitigate code multipath errors. A code multipath model based on experimental CMC data from different environments was established in [20]. However, CMC only reflects the composite effect of all multipath signals and cannot resolve individual paths or estimate specific multipath parameters, such as delay, attenuation, and lifetime. To accurately compute the parameters of each path, ray-tracing techniques are employed to obtain all potential multipath signals within a specific scenario [21]. Nevertheless, this method requires a priori precise 3D maps, making it unsuitable for unknown environments. The Okumura model [22], a statistical multipath channel model for wireless communications, is widely used for predicting signal path loss. The study in [23] utilized the Multipath Estimating Delay Lock Loop (MEDLL) to achieve Time-of-Arrival (TOA) estimation for LTE signals in multipath environments, outlining the process for estimating parameters of the detected multipath components. Research in [24] investigated multipath channel estimation and dynamic channel statistical modeling, including the number of multipath components, their delays, amplitudes, Doppler fading frequencies, component lifetimes, and Non-Line-of-Sight (NLOS) signal detection.

### 2.3. Multipath Estimation Based on Object Tracking

In indoor positioning based on multipath estimation, many researchers utilize the concept of Virtual Anchors (VAs) to model the surrounding environment. A virtual anchor is the mirror image of a physical anchor across a reflective surface. These approaches transform the multipath estimation problem into an anchor target tracking problem. The estimated states of the positioning terminal and the virtual anchors are represented by time-varying marginal distributions. Reference [25] formulates the joint probability density function (PDF) for a single snapshot as a factor graph and employs a message-passing algorithm to compute the marginal distributions. The BP-SLAM algorithm proposed in [11] probabilistically models the states of both physical and virtual anchors, along with data association uncertainty, using delay parameters extracted from Ultra-Wideband (UWB) signals. Furthermore, reference [26] proposed jointly estimating the mobile agent’s position, orientation, and clock offset, as well as the state of the surrounding environment, using Time of Arrival (TOA), Angle of Arrival (AOA), and Angle of Departure (AOD). Reference [27] employed the concept of a unique master virtual anchor, modeling only single reflections without estimating all virtual anchors. To leverage temporal information in state estimation, reference [28] introduced a novel Bayesian model, BP-CC, which considers measurements from previous snapshots to jointly estimate the agent’s state and its surrounding environment.

## 3. LiDAR Point Cloud-Based Reflective Surface Detection Methods

**Figure 2:**
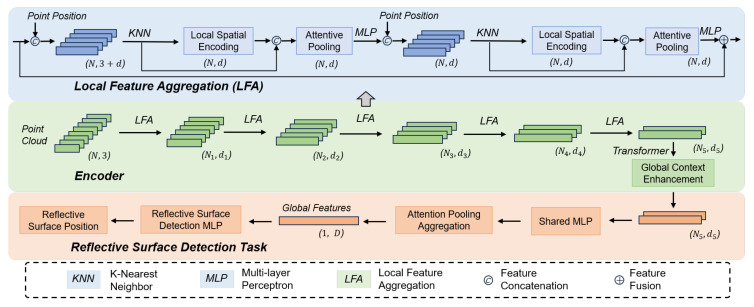
The neural network model for detecting reflection surfaces with semantic correlation is composed of an encoder layer (semantic feature extraction layer) and a reflection surface detection layer.

LiDAR point clouds contain rich environmental perception information. Therefore, this paper proposes a LiDAR point cloud-based method for detecting reflective surface positions. LiDAR point clouds offer high resolution and can extract substantial semantic information, including the shapes and locations of potential reflective surfaces such as doors, windows, and walls. Building on this, we utilize LiDAR point clouds to extract semantic information, thereby enhancing reflective surface detection capability. To fully leverage the semantic information, we constructed a semantically associated neural network model for reflective surface detection, as shown in Figure 2.

The model comprises an encoder layer (semantic feature extraction layer) and a reflective surface detection layer. First, the encoder layer is responsible for semantic feature extraction. It extracts extensive semantic information by stacking downsampling and local feature aggregation. Local feature aggregation is applied in parallel to each point [29] and primarily consists of three neural units: local spatial encoding, attentive pooling, and dilated residual blocks. Local spatial encoding explicitly embeds the 3D coordinates of all K-nearest neighboring points, ensuring the corresponding point features are always aware of their relative spatial positions. Attentive pooling utilizes an attention mechanism to learn local features. The dilated residual block incorporates skip connections inspired by ResNet to preserve geometric details. The values $N_{1}$ to $N_{5}$ are 40,960, 10,240, 2560, 640, and 160, respectively. The values $d_{1}$ to $d_{5}$ are 16, 64, 128, 256, and 512, respectively. Subsequently, the reflective surface detection layer is responsible for outputting the shape and positional information of potential reflective surfaces. It aggregates high-dimensional semantic features along with the semantic segmentation information of these points. Since we only consider reflective surfaces perpendicular to the ground, these surfaces can be represented as line segments in the top-down view. The output potential reflective surface information is formatted into $M_{pre}$ fixed slots. The first dimension of each slot is the reflective surface existence confidence, representing the probability of the surface’s presence. The remaining dimensions are the coordinates of the reflective surface’s center point $\left(x_{refl},y_{refl}\right)$, its length len, and the unit normal vector of the surface $n_{refl}=\left(n_{x},n_{y}\right)$. The center point coordinates and the unit normal vector are used to describe the position of the reflective surface. The length is used in the next chapter to assess the existence of multipath reflections from anchors.

Since the number of output slots is fixed, while the actual number of true labels $M_{label}$ is variable, each slot may correspond to a reflective surface position. However, the correspondence between these predicted surfaces and the training labels is unknown. Therefore, we employ Hungarian matching to solve the optimal one-to-one assignment problem between predictions and ground truth, ensuring effective training. When constructing the cost matrix $C_{M_{pre}\times M_{label}}$, each element $C_{i,j}$ is defined as

(1) $$C_{i,j}=C_{pos}\left(i,j\right)+C_{len}\left(i,j\right)+C_{n}\left(i,j\right)$$

where $C_{pos}\left(i,j\right)$ represents the regression cost between the center coordinates of the predicted reflective surface *i* and the ground truth reflective surface *j*. $C_{len}\left(i,j\right)$ denotes the regression cost for the length of the reflective surface, and $C_{n}\left(i,j\right)$ indicates the regression cost for the surface’s normal vector. All are computed using the Smooth L1 Loss.

The reflective surface detection loss function $L_{refl}$ is composed as shown in (2):

(2) $$L_{refl}=L_{conf}+L_{cen}+L_{len}+L_{n}$$

where $L_{conf}$ denotes the confidence loss, $L_{cen}$ represents the reflective surface center position loss, $L_{len}$ is the reflective surface length loss, and $L_{n}$ corresponds to the reflective surface inclination angle loss. We use $\hat{\left(\cdot \right)}$ to denote the predicted value. Each loss function is defined as follows:

(3) $$L_{conf}=-\frac{1}{M_{pre}}\sum_{i}\left[{\hat{p}}_{i}\log\left(p_{i}\right)+\left(1-{\hat{p}}_{i}\right)\log\left(1-p_{i}\right)\right]$$

(4) $$L_{cen}=\frac{1}{M_{match}}\sum_{i}\frac{\mathrm{Smooth}\;L1\left({\hat{x}}_{refl},x_{refl}\right)+\mathrm{Smooth}\;L1\left({\hat{y}}_{refl},y_{refl}\right)}{\hat{len}}$$

(5) $$L_{len}=\frac{1}{M_{match}}\sum_{i}\frac{\mathrm{Smooth}\;L1(\hat{len},len)}{\hat{len}}$$

(6) $$L_{n}=\frac{1}{M_{match}}\sum_{i}\left(1-\frac{{\hat{n}}_{refl}\cdot n_{refl}}{∥{\hat{n}}_{refl}∥∥n_{refl}∥}\right)$$

where $M_{match}$ denotes the number of reflective surfaces after optimal matching between the predicted and ground truth surfaces. We only utilize the successfully matched reflective surface positions to calculate the loss function. The slots that are not matched are considered to have no reflective surface present.

The output of the reflective surface detection is denoted as

(7) $$l_{k}={\left[p_{refl,k},P_{l,k}^{T},len_{k},n_{refl}\right]}^{T}$$

where k denotes the index of candidate reflective surfaces detected by LiDAR, with a value range of $1\leq k\leq K_{n}$ ($K_{n}$ is the total number of candidate reflective surfaces in the n-th epoch), $p_{refl,k}$ represents the detection probability of the reflective surface, $P_{l,k}^{T}$ denotes its center position point, $len_{k}$ indicates the length of the surface, and $n_{refl}$ is the normal vector of the reflective surface.

## 4. Factor Graph-Based Multipath Consistency Checking and Localization Method

The indoor positioning environment is complex, with few direct paths from the signal transmission anchors to the mobile terminal. Consequently, relying solely on the limited anchors identified as LOS through NLOS recognition results in a low positioning accuracy. The signal measurements received by the terminal contain a significant amount of multipath propagation. Virtual anchor positioning methods can utilize multipath information to increase the effective number of anchors. Simultaneously, LiDAR can perceive potential reflective surfaces in the environment, which provides geometric constraints for the locations of these virtual anchors. To address this, we propose a factor graph-based multipath consistency verification and localization method. This method establishes a three-dimensional association between signal measurements, semantically perceived reflective surfaces, and historical virtual anchor states, thereby enhancing the reliability of the utilized multipath information. Since this method involves a large number of symbols, we have compiled the frequently used ones in Table 1 for easy reference.

### 4.1. System Model

We consider a two-dimensional positioning scenario in an indoor environment involving a mobile terminal and several signal anchors. At epoch *n*, the terminal’s position is denoted as $p_{n}$, and it receives *J* signals from physical anchors located at $p_{ra}^{j}$, j∈{1,…,J}. Each physical anchor transmits wireless signals to the terminal, which arrive via the LOS path and multiple reflection paths. Due to significant signal attenuation caused by multiple reflections, we consider only single-bounce scenarios. Assuming there are *S* reflective surfaces in the environment, the position of the virtual anchor corresponding to the *s* surface for the *j* anchor is denoted as $p_{va,s}^{j}$, s∈{1,…,S}. The relationship between a virtual anchor and its corresponding physical anchor is given by:

(8) $$p_{va,s}^{j}=p_{ra}^{j}+2\left(u_{s}^{T}P_{s}-u_{s}^{T}p_{ra}^{j}\right)u_{s}$$

Here, $u_{s}^{T}$ is the normal vector of the reflective surface, and $P_{s}$ is a point on that surface. To facilitate the description of the relationship between the virtual anchor and the reflective surface, we define the point corresponding to the origin in the reflective surface as the Virtual Base Anchor (VBA) $p_{vba,s}$, as given in [30]. Each VBA corresponds to one reflective surface. Therefore, in the following text, we use the VBA to represent the reflective surface. Based on the LiDAR perception result $l_{k}$ and the center point $P_{l,k}^{T}$ of the detected reflective surface, the LiDAR-perceived VBA $p_{vba,k}$ can be calculated by (8). The relationship between the VBA and the virtual anchor is given by:

(9) $$p_{va,s}^{j}=-\left(\frac{2\langle p_{vba,s},p_{ra}^{j}\rangle }{{∥p_{vba,s}∥}^{2}}-1\right)p_{vba,s}+p_{ra}^{j}$$

where 〈·〉 denotes the vector inner product.

The state of the terminal is defined as $x_{n}=\left[\begin{array}{c} p_{n}^{T}\;v_{n}^{T} \end{array}\right]$, where $v_{n}^{T}$ is the velocity component. The state of a reflective surface is defined as $y_{n}={\left[p_{vba,s}^{T}\;r_{n,s}\right]}^{T}$, where $r_{n,s}\in \left\{0,1\right\}$ is a binary state variable: 0 indicates the reflective surface does not exist, and 1 indicates it exists. This variable $r_{n,s}$ is introduced to account for the constraint imposed by the reflective surface on the observations under both its existence and non-existence scenarios.

Assuming the terminal receives $M_{n}^{j}$ multipath components from the signal transmitted by anchor *j*, the observation model for the received signal on each arriving path is:

(10) $$z_{m,n}^{j}=∥p_{va,s}^{j}-p_{n}∥+v_{m,n}^{j},\;m\in \left\{1,\ldots ,M_{n}^{j}\right\}$$

Here, $v_{m,n}^{j}$ is the zero-mean Gaussian measurement noise with variance $\sigma_{m,n}^{j}$. The multipath components can be analyzed and obtained via the super-resolution MUSIC algorithm [5]. Thus, the likelihood function for the Line-of-Sight (LOS) path is given by:

(11) $$f\left(z_{m,n}^{j}|p_{n}\right)=\frac{1}{\sqrt{2\pi {\left(\sigma_{m,n}^{j}\right)}^{2}}}\exp\left(-\frac{{\left(z_{m,n}^{j}-∥p_{ra}^{j}-p_{n}∥\right)}^{2}}{2{\left(\sigma_{m,n}^{j}\right)}^{2}}\right)$$

Assuming that the signal measurement process and the LiDAR perception process are independent, the likelihood function for the reflected path is given by:

(12) $$f\left(z_{m,n}^{j},l_{k}|p_{n},p_{va,s}^{j}\right)=f\left(z_{m,n}^{j}|p_{n},p_{va,s}^{j}\right)*f\left(p_{vba,k}|p_{va,s}^{j}\right)$$

where $p_{vba,k}$ is the LiDAR-perceived VA position calculated by (9). $f\left(p_{vba,k}|p_{va,s}^{j}\right)$ is also considered a Gaussian distribution with variance $\sigma_{k,n}^{j}$. The newly detected VBAs at each epoch can be modeled as a Poisson point process [30] with mean $\mu_{n}$ and probability distribution $f_{n}\left(p_{vba,m}^{j}|p_{n}\right)$. $f_{n}$ is set as a uniform distribution over the field area, and $\mu_{n}$ is set to 0.05. For each newly measured multipath component $z_{m,n}^{j}$, the state of its corresponding VBA is denoted as ${\bar{y}}_{n,m}^{j}$, treated as a candidate reflective surface. The previously propagated VBA states are denoted as ${\underline{y}}_{n,s}^{j}$. The state transition process is carried out sequentially across different epochs and different anchors. For epoch *n* and anchor *j*, all measurements from epochs $n^{\prime }<n$ and from anchors $j^{\prime }<j$ at the current epoch are considered as prior inheritances. Therefore, during different anchor state transition, the total number of existing VBAs is updated as $S_{n}^{j}=S_{n}^{j-1}+M_{n}^{j-1}$.

Since the positions of reflective surfaces are fixed, during VBA state transition, only ${\underline{r}}_{n,s}^{j}$ concerned, whether the reflective surface is retained—is considered. We incorporate temporal state transition at the first base station of each epoch. A fading factor λ (set to 0.99) is used to represent the retention probability of the reflective surface across epochs. When the VBA exists,

(13) $$f\left({\underline{y}}_{n,s}^{1}|{\underline{p}}_{vba,s}^{1},{\underline{r}}_{n-1,s}^{1}=1\right)=\left\{\begin{array}{cc} \left(1-\lambda \right)f_{d}\left({\underline{p}}_{vba,s}^{1}\right), & {\underline{r}}_{n,s}^{1}=0\\ \lambda \delta \left({\underline{p}}_{vba,s}^{1}-p_{vba,s}^{1}\right), & {\underline{r}}_{n,s}^{1}=1 \end{array}\right.$$

where $f_{d}\left(\cdot \right)$ is a uniform distribution function, indicating that the VBA position is arbitrary when the anchor does not exist. We set it to the average value of the field area. $\delta_{d}\left(\cdot \right)$ is the Dirac delta function, representing that the reflective surface position remains unchanged.

When the VBA does not exist, the state transition is defined as:

(14) $$f\left({\underline{y}}_{n,s}^{1}|{\underline{p}}_{vba,s}^{1},{\underline{r}}_{n-1,s}^{1}=0\right)=\left\{\begin{array}{cc} f_{d}\left({\underline{p}}_{vba,s}^{1}\right), & {\underline{r}}_{n,s}^{1}=0\\ 0, & {\underline{r}}_{n,s}^{1}=1 \end{array}\right.$$

For other base stations within the same epoch, the state transition between base stations is defined as:

(15) $$f\left({\underline{y}}_{n,s}^{j}|{\underline{p}}_{vba,s}^{j-1},{\underline{r}}_{n,s}^{j-1}=1\right)=\left\{\begin{array}{cc} f_{d}\left({\underline{p}}_{vba,s}^{j-1}\right), & {\underline{r}}_{n,s}^{j}=0\\ \delta \left({\underline{p}}_{vba,s}^{j}-p_{vba,s}^{j-1}\right), & {\underline{r}}_{n,s}^{j}=1 \end{array}\right.$$

(16) $$f\left({\underline{y}}_{n,s}^{j}\;|\;{\underline{p}}_{vba,s}^{j-1},{\underline{r}}_{n,s}^{j-1}=0\right)=\left\{\begin{array}{c} \begin{array}{ccc} f_{d}\left({\underline{p}}_{vba,s}^{j}\right), & {\underline{r}}_{n,s}^{1} & =0\\ 0, & {\underline{r}}_{n,s}^{1} & =1 \end{array} \end{array}\right.$$

### 4.2. Multi-Dimensional Data Association

There exists an uncertain association between the LiDAR-perceived reflective surfaces, the actual reflective surfaces derived from signal measurements, and the reflective surfaces from previous epochs. This uncertainty directly constrains the confirmation of multipath information. Therefore, an association variable *a* is introduced to describe the relationship between the current epoch’s measurement $z_{m,n}^{j}$, the LiDAR-perceived reflective surface $l_{k,n}$, and the VBAs from prior epochs. Since only single-bounce reflections are considered, for each anchor, one VBA can generate at most one multipath measurement, and one multipath component originates from only one VBA. Consequently, the value space of the association variable is discrete. We use ${\underline{a}}_{s}^{j}$ to denote the association variable for VBAs carried over from previous epochs and anchors. Its value space is the set of currently observed VBAs: ${\underline{a}}_{s}^{j}\in \left\{0,\left(1,0\right),\left(1,1\right),\ldots ,\left(M_{n}^{j},K_{n}\right)\right\}$. Here, 0 indicates that the *s* prior VBA from anchor *j* does not correspond to any reflective surface in the current epoch. The pair (m,k) signifies that this VBA generated the current epoch’s multipath measurement $z_{m,n}^{j}$ and was perceived by LiDAR as $l_{k,n}$. The pair (m,0) indicates that this VBA generated the multipath measurement $z_{m,n}^{j}$ but was not perceived by LiDAR (potentially corresponding to a LOS path). Simultaneously, we use ${\bar{a}}_{m,k}^{j}$ to represent the association variable for the VBA observed in the current epoch for the current anchor. Its value space is the set of prior VBAs. ${\bar{a}}_{m,k}^{j}\in \left\{0,1,\ldots ,S_{j}\right\}$. Here, 0 indicates that the *m* newly observed VBA for anchor *j*, perceived by LiDAR as $l_{k}$, does not correspond to any prior VBA. A value *s* (where s>0) signifies that this new VBA was generated by the prior VBA.

### 4.3. Factor Graph-Based Estimation Process

#### 4.3.1. Estimated State

The states we estimate include the state of the mobile terminal and the states of the reflective surfaces. The estimation process for the mobile terminal state can be described as:

(17) $${\hat{x}}_{n}\leftarrow \int {\hat{x}}_{n}f\left(x_{n}|z_{1:n},l_{1:n}\right)dx_{n}$$

where $z_{1:n}={\left[z_{1}^{T},\ldots ,z_{n}^{T}\right]}^{T}$, $z_{n}={\left[{\left(z_{n}^{1}\right)}^{T},\ldots ,{\left(z_{n}^{J}\right)}^{T}\right]}^{T}$, $z_{n}^{j}={\left[{\left(z_{1,n}^{j}\right)}^{T},\ldots ,{\left(z_{M_{n}^{j},n}^{j}\right)}^{T}\right]}^{T}$, $l_{1:n}={\left[l_{1}^{T},\ldots ,l_{n}^{T}\right]}^{T}$, and $l_{n}={\left[{\left(l_{1,n}\right)}^{T},\ldots ,{\left(l_{K_{n},n}\right)}^{T}\right]}^{T}$, representing the information from all previous epochs.

The estimation process for the state of the reflective surface represented by the VBA can be described as:

(18) $${\hat{p}}_{\mathrm{vba},s}\leftarrow \int p_{\mathrm{vba},s}f\left(p_{\mathrm{vba},s}|r_{n,s}=1,z_{1:n},l_{1:n}\right)dp_{\mathrm{vba},s}$$

where $f(p_{\mathrm{vba},s}|r_{n,s}=1,z_{1:n},l_{1:n})$ is obtained from the marginal posterior probability distribution:

(19) $$f\left(p_{\mathrm{vba},s}|r_{n,s}=1,z_{1:n},l_{1:n}\right)=\frac{f(p_{\mathrm{vba},s},r_{n,s}=1|z_{1:n},l_{1:n})}{\int f\left(p_{\mathrm{vba},s},r_{n,s}=1|z_{1:n},l_{1:n}\right)dp_{\mathrm{vba},s}}$$

#### 4.3.2. Factor Graph Design

**Figure 3:**
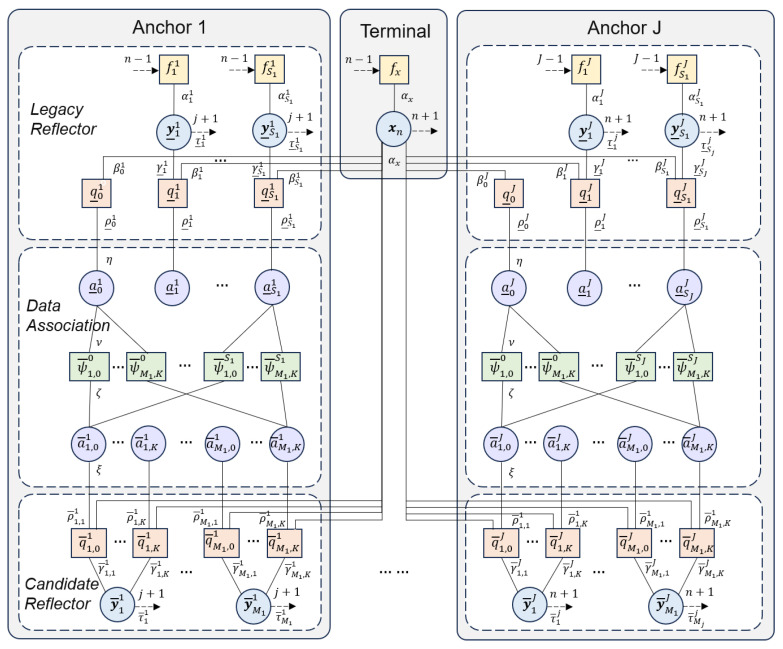
Factor graph of potential reflection surface and multipath estimation.

The factor graph for potential reflective surfaces and multipath estimation is shown in Figure 3. We referenced the multipath-SLAM factor graph structure in [30], and on this basis, we incorporated the association of LiDAR perception information. We decompose the joint posterior probability distribution *f* of the factor graph into the prior initial state factor $\phi_{\mathrm{ini}}$, the terminal state transition factor $\phi_{ts-trans}$, the anchor state transition factor $\phi_{as-trans}$, the LOS path factor $\phi_{d}$, the residual VBA reflection path factor $\phi_{legacy-vba}$, and the new VBA factor $\phi_{new-vba}$ to give

(20) $$f\left(y_{0:n},x_{0:n},{\underline{a}}_{1:n},{\bar{a}}_{1:n}\;|\;z_{1:n},l_{1:n}\right)\propto \phi_{\mathrm{ini}}\times \phi_{\mathrm{ts}-\mathrm{trans}}\times \phi_{\mathrm{as}-\mathrm{trans}}\times \phi_{d}\times \phi_{\mathrm{legacy}-\mathrm{vba}}\times \phi_{\mathrm{new}-\mathrm{vba}}$$

(21) $$\phi_{\mathrm{ini}}=\left(f\left(x_{0}\right)\prod_{s=1}^{S_{0}}f\left(y_{0,s}\right)\right)$$

(22) $$\phi_{\mathrm{ts}-\mathrm{trans}}=\prod_{n^{\prime }=1}^{n}f\left(x_{n^{\prime }},x_{n^{\prime }-1}\right)$$

(23) $$\phi_{\mathrm{as}-\mathrm{trans}}=\left(\prod_{s^{\prime }=1}^{S_{n^{\prime }-1}}f\left({\underline{y}}_{n^{\prime },s^{\prime }},y_{n^{\prime }-1,s^{\prime }}\right)\right)\times \left(\prod_{j^{\prime }=2}^{J}\left(\prod_{s^{\prime }=1}^{S_{n^{\prime }}^{j^{\prime }}}f\left({\underline{y}}_{n^{\prime },s^{\prime }}^{j^{\prime }},{\underline{y}}_{n^{\prime },s^{\prime }}^{j^{\prime }-1}\right)\right)\right)$$

(24) $$\phi_{d}=\left(\prod_{j=1}^{J}{\underline{q}}_{D}\left(x_{n^{\prime }},{\underline{a}}_{0,n^{\prime }}^{j};z_{n^{\prime }}^{j}\right)\prod_{m^{\prime }=1}^{M_{n^{\prime }}^{j}}\prod_{k^{\prime }=0}^{K_{n^{\prime }}}\Psi \left({\underline{a}}_{0,n^{\prime }}^{j},{\bar{a}}_{\left(m^{\prime },k^{\prime }\right),n^{\prime }}^{j}\right)\right)$$

(25) $$\phi_{legacy-vba}=\prod_{j=1}^{J}\left(\prod_{s^{\prime }=1}^{S_{n^{\prime }}^{j}}{\underline{q}}_{R}\left({\underline{y}}_{n^{\prime },s^{\prime }}^{j},{\underline{a}}_{s^{\prime },n^{\prime }}^{j},x_{n^{\prime }};z_{n^{\prime }}^{j},l_{n^{\prime }}\right)\right)\prod_{m^{\prime }=1}^{M_{n^{\prime }}^{j}}\left(\prod_{k^{\prime }=0}^{K_{n^{\prime }}}\Psi \left({\underline{a}}_{s^{\prime },n^{\prime }}^{j},{\bar{a}}_{\left(m^{\prime },k^{\prime }\right),n^{\prime }}^{j}\right)\right)$$

(26) $$\phi_{new-vba}=\left(\prod_{j=1}^{J}\prod_{m=1}^{M_{n^{\prime }}^{j}}\prod_{k^{\prime }=0}^{K_{n^{\prime }}}{\bar{q}}_{R}\left({\bar{y}}_{n^{\prime },m}^{j},{\bar{a}}_{m,n^{\prime }}^{j},x_{n^{\prime }};z_{n^{\prime }}^{j},l_{n^{\prime }}\right)\right)$$

where ${\underline{q}}_{D}\left(x_{n},{\underline{a}}_{0,n}^{j};z_{n}^{j}\right)$ represents the pseudo-likelihood function for the direct path from the anchor. When ${\underline{a}}_{0,n}^{j}=\left(m,k\right),m\in M_{n}^{j}$, then ${\underline{q}}_{D}\left(x_{n},{\underline{a}}_{0,n}^{j};z_{n}^{j}\right)=p_{rt}^{j}\left(p_{n}\right)f\left(z_{m,n}^{j}|p_{n}\right)$; when ${\underline{a}}_{0,n}^{j}=0$, then ${\underline{q}}_{D}\left(x_{n},{\underline{a}}_{0,n}^{j};z_{n}^{j}\right)=1-p_{rt}^{j}\left(p_{n}\right)$. Here, $p_{rt}^{j}\left(p_{n}\right)$ relies on geometric constraints from LiDAR point clouds. Specifically, $p_{rt}^{j}\left(p_{n}\right)=0$ when point cloud occlusion exists between the anchor and terminal; otherwise, it equals 1.

${\underline{q}}_{R}\left({\underline{y}}_{n,s}^{j},{\underline{a}}_{s,n}^{j},x_{n};z_{n}^{j},l_{n}\right)$ represents the pseudo-likelihood function for the reflected path from the historical VBA. First, considering the case where the reflective surface exists, if ${\underline{a}}_{s,n}^{j}=\left(m,k\right),m\in M_{n}^{j}$, then

(27) $${\underline{q}}_{R}\left({\underline{p}}_{vba,s}^{j},{\underline{r}}_{n,s}^{j}=1,{\underline{a}}_{s,n}^{j},x_{n};z_{n}^{j},l_{n}\right)=p_{rt}^{j}\left(p_{n},{\underline{p}}_{vba,s}^{j}\right)f\left(z_{m,n}^{j},l_{k}|p_{n},{\underline{p}}_{vba,s}^{j}\right)$$

otherwise, ${\underline{q}}_{R}\left({\underline{p}}_{\mathrm{vba},s}^{j},{\underline{r}}_{n,s}^{j}=1,{\underline{a}}_{s,n}^{j},x_{n};z_{n}^{j},l_{n}\right)=1-p_{\mathrm{rt}}^{j}\left(p_{n},{\underline{p}}_{\mathrm{vba},s}^{j}\right)$. Next, consider the case where the reflective surface does not exist. If ${\underline{a}}_{s,n}^{j}=\left(m,k\right),m\in M_{n}^{j}$, then ${\underline{q}}_{R}\left({\underline{p}}_{\mathrm{vba},s}^{j},{\underline{r}}_{n,s}^{j}=0,{\underline{a}}_{s,n}^{j},x_{n};z_{n}^{j},l_{n}\right)=0$; otherwise, if ${\underline{a}}_{s,n}^{j}=0$, then ${\underline{q}}_{R}\left({\underline{p}}_{\mathrm{vba},s}^{j},{\underline{r}}_{n,s}^{j}=0,{\underline{a}}_{s,n}^{j}=0,x_{n};z_{n}^{j},l_{n}\right)=1$.

${\bar{q}}_{R}\left({\bar{y}}_{n,m},{\bar{a}}_{(m,k),n}^{j},x_{n};z_{n}^{j},l_{n}\right)$ represents the pseudo-likelihood function for the newly observed VBA. First, consider the case where the reflective surface exists. When ${\bar{a}}_{(m,k),n}^{j}=s,s\in S_{n}^{j}$, it indicates that it coincides with a previous VBA, so ${\bar{q}}_{R}\left({\bar{p}}_{\mathrm{vba},m}^{j},{\bar{r}}_{n,m}^{j}=1,{\bar{a}}_{(m,k),n}^{j},x_{n};z_{n}^{j},l_{n}\right)=0$. Only when ${\bar{a}}_{(m,k),n}^{j}=0$, meaning it is not associated with any historical VBA,

(28) $${\bar{q}}_{R}\left({\bar{p}}_{vba,m}^{j},{\bar{r}}_{n,m}^{j}=1,{\bar{a}}_{(m,k),n}^{j},x_{n};z_{n}^{j},l_{n}\right)=\mu_{n}f_{n}\left({\bar{p}}_{vba,m}^{j}|p_{n}\right)f\left(z_{m,n}^{j},l_{k}|p_{n},{\bar{p}}_{vba,m}^{j}\right)$$

Next, consider the case where the reflective surface does not exist: ${\bar{q}}_{R}\left({\bar{p}}_{\mathrm{vba},m}^{j},{\bar{r}}_{n,m}^{j}=0,{\bar{a}}_{(m,k),n}^{j},x_{n};z_{n}^{j},l_{n}\right)=f_{d}\left({\bar{p}}_{\mathrm{vba},m}^{j}\right)$.

$\Psi ({\underline{a}}_{s,n}^{j},{\bar{a}}_{(m,k),n}^{j})$ denotes the association validation function. It equals 1 only when ${\underline{a}}_{s,n}^{j}=\left(m,k\right)$ and ${\bar{a}}_{(m,k),n}^{j}=s$; otherwise, it equals 0. The purpose is to enforce a mutual association between historical and new VBAs, avoiding one-to-many mappings.

### 4.4. The Calculation Process of Factor Graph

The message-passing process in the factor graph includes: temporal state prediction of the terminal and reflective surfaces, state transition and update between anchors, message passing of signal measurement and LiDAR perception constraints, data association, state update messages, and final state estimation. Temporal state prediction is performed at the first anchor of each epoch, while state transition is executed for the other anchors. The computational procedures for each step are as follows.

#### 4.4.1. Temporal State Prediction for Terminal and Reflective Surfaces

The state prediction message for the terminal is:

(29) $$\alpha \left(x_{n}\right)=\int f\left(x_{n}|x_{n-1}\right)\hat{f}\left(x_{n-1}\right)dx_{n-1}$$

The temporal state prediction message for a prior reflective surface is:

(30) $$\alpha \left({\underline{p}}_{\mathrm{vba},s}^{1},{\underline{r}}_{n,s}^{1}\right)=\sum_{r_{n-1,s}=0,1}\int f\left({\underline{p}}_{\mathrm{vba},s}^{1},{\underline{r}}_{n,s}^{1}|p_{\mathrm{vba},s},r_{n-1,s}\right)\hat{f}\left(p_{\mathrm{vba},s},r_{n-1,s}\right)dp_{\mathrm{vba},s}$$

where $p_{\mathrm{vba},s},r_{n-1,s}$ represents the VBA state after the update from the last anchor of the previous epoch.

#### 4.4.2. State Transition and Update Between Anchors

For other anchors in the current epoch, the state transition message is:

(31) $$\alpha \left({\underline{p}}_{\mathrm{vba},s}^{j},{\underline{r}}_{n,s}^{j}\right)=\sum_{{\underline{r}}_{n,s}=0,1}\int f\left({\underline{p}}_{\mathrm{vba},s}^{j},{\underline{r}}_{n,s}^{j}|{\underline{p}}_{\mathrm{vba},s}^{j-1},{\underline{r}}_{n,s}^{j-1}\right)\hat{f}\left({\underline{p}}_{\mathrm{vba},s}^{j-1},{\underline{r}}_{n,s}^{j-1}\right)d{\underline{p}}_{\mathrm{vba},s}^{j-1}$$

#### 4.4.3. Message Passing for Signal Measurement and LiDAR Perception Constraints

The message passing for the direct path is:

(32) $${\underline{\rho }}_{0}\left({\underline{a}}_{0,n}^{j}\right)=\int \alpha \left(x_{n}\right){\underline{q}}_{D}\left(x_{n},{\underline{a}}_{0,n}^{j};z_{n}^{j}\right)dx_{n}$$

The message passing for prior VBAs is:

(33) $${\underline{\rho }}_{s}\left({\underline{a}}_{s,n}^{j}\right)=\sum_{{\underline{r}}_{n,s}^{j}=0,1}\;\int \int \;\alpha \left(x_{n}\right)\alpha \left({\underline{p}}_{\mathrm{vba},s}^{j},{\underline{r}}_{n,s}^{j}\right){\underline{q}}_{R}\left({\underline{y}}_{n,s}^{j},{\underline{a}}_{s,n}^{j},x_{n};z_{n}^{j},l_{n}\right)dx_{n}d{\underline{p}}_{\mathrm{vba},s}^{j}$$

The message passing for new VBAs is:

(34) $${\bar{\rho }}_{s}\left({\bar{a}}_{(m,k),n}^{j}\right)=\sum_{{\bar{r}}_{n,m}^{j}=0,1}\;\int \int \;\alpha \left(x_{n}\right)\alpha \left({\underline{p}}_{\mathrm{vba},s}^{j},{\underline{r}}_{n,s}^{j}\right){\bar{q}}_{R}\left({\bar{y}}_{n,m},{\bar{a}}_{(m,k),n}^{j},x_{n};z_{n}^{j},l_{n}\right)dx_{n}d{\bar{p}}_{\mathrm{vba},m}^{j}$$

#### 4.4.4. Data Association

The data association message from new VBAs to prior VBAs is:

(35) $$\eta \left({\underline{a}}_{s,n}^{j}\right)=\prod_{m=1}^{M_{n}^{j}}\prod_{k=0}^{K_{n}}\upsilon_{(m,k)\to s}\left({\underline{a}}_{s,n}^{j}\right)$$

The data association message from prior VBAs to new VBAs is:

(36) $$\xi \left({\bar{a}}_{(m,k),n}^{j}\right)=\prod_{s=0}^{S_{n}^{j}}\zeta_{s\to (m,k)}\left({\bar{a}}_{(m,k),n}^{j}\right)$$

where the messages $\upsilon_{(m,k)\to s}$ and $\zeta_{s\to (m,k)}$ are obtained through an iterative process [30].

#### 4.4.5. State Update Messages

The terminal update message based on signal measurements and LiDAR perception is:

(37) $$\beta_{0}^{j}\left(x_{n}\right)=\sum_{{\underline{a}}_{0,n}^{j}=\left(1,0\right)}^{\left(M_{n}^{j},K_{n}\right)}\eta \left({\underline{a}}_{0,n}^{j}\right){\underline{q}}_{D}\left(x_{n},{\underline{a}}_{0,n}^{j};z_{n}^{j}\right)$$

(38) $$\beta_{s}^{j}\left(x_{n}\right)=\sum_{{\underline{a}}_{s,n}^{j}=\left(1,0\right)}^{\left(M_{n}^{j},K_{n}\right)}\sum_{r_{n,s}=0,1}\eta \left({\underline{a}}_{0,n}^{j}\right)\int \alpha \left(x_{n}\right){\underline{q}}_{R}\left({\underline{y}}_{n,s}^{j},{\underline{a}}_{s,n}^{j},x_{n};z_{n}^{j},l_{n}\right)d{\underline{p}}_{\mathrm{vba},s}^{j}$$

The update message for the prior VBA is:

(39) $${\underline{\gamma }}_{s}\left({\underline{y}}_{n,s}^{j}\right)=\sum_{{\underline{a}}_{s,n}^{j}=\left(1,0\right)}^{\left(M_{n}^{j},K_{n}\right)}\eta \left({\underline{a}}_{s,n}^{j}\right)\int \alpha \left(x_{n}\right){\underline{q}}_{R}\left({\underline{y}}_{n,s}^{j},{\underline{a}}_{s,n}^{j},x_{n};z_{n}^{j},l_{n}\right)dx_{n}$$

(40) $${\underline{\tau }}_{s}^{j}\left({\underline{y}}_{n,s}^{j}\right)=\alpha \left({\underline{y}}_{n,s}^{j}\right){\underline{\gamma }}_{s}\left({\underline{y}}_{n,s}^{j}\right)$$

The update message for the new VBA is:

(41) $${\bar{\gamma }}_{m,k}\left({\bar{y}}_{n,m}^{j}\right)=\sum_{{\bar{a}}_{(m,k),n}^{j}=0}^{S_{n}^{j}}\xi \left({\bar{a}}_{(m,k),n}^{j}\right)\int \alpha \left(x_{n}\right){\bar{q}}_{R}\left({\bar{y}}_{n,m},{\bar{a}}_{(m,k),n}^{j},x_{n};z_{n}^{j},l_{n}\right)dx_{n}$$

(42) $${\bar{\tau }}_{m,k}^{j}\left({\bar{y}}_{n,m}^{j}\right)=\prod_{m=1}^{M_{n}^{j}}{\bar{\gamma }}_{m,k}\left({\bar{y}}_{n,m}^{j}\right)$$

#### 4.4.6. Final State Estimation

The final state estimate for the terminal is obtained by taking the product of all messages and normalizing:

(43) $$\hat{f}\left(x_{n}\right)=\frac{\alpha \left(x_{n}\right)\left(\prod_{s=0}^{S_{n}^{j}}\beta_{s}^{J}\left(x_{n}\right)\right)\left(\prod_{j=1}^{J}\beta_{0}^{j}\left(x_{n}\right)\right)}{\int \alpha \left(x_{n}\right)\left(\prod_{s=0}^{S_{n}^{j}}\beta_{s}^{J}\left(x_{n}\right)\right)\left(\prod_{j=1}^{J}\beta_{0}^{j}\left(x_{n}\right)\right)dx_{n}}$$

The final state estimate for a prior VBA is given by:

(44) $$\hat{f}\left({\underline{y}}_{n,s}^{J}\right)=\frac{{\underline{\tau }}_{s}^{j}\left({\underline{y}}_{n,s}^{j}\right)}{\sum_{r_{n,s}=0,1}\int {\underline{\tau }}_{s}^{j}\left({\underline{p}}_{\mathrm{vba},s}^{j},r_{n,s}\right)d{\underline{p}}_{\mathrm{vba},s}^{j}}$$

The final state estimate for a new VBA is given by:

(45) $$\hat{f}\left({\bar{y}}_{n,m}^{J}\right)=\frac{{\bar{\tau }}_{m,k}^{j}\left({\bar{y}}_{n,m}^{j}\right)}{\sum_{r_{n,m}=0,1}\int {\bar{\tau }}_{m,k}^{j}\left({\bar{p}}_{\mathrm{vba},m}^{j},r_{n,m}\right)d{\bar{p}}_{\mathrm{vba},m}^{j}}$$

To prevent the computational load from becoming excessive as the number of prior VBAs $S_{n}^{j}$ increases over time, a threshold $P_{\mathrm{th}}$ is set. A VBA is pruned if its retention probability falls below the threshold $P_{\mathrm{th}}$, which is set to 0.01. Furthermore, a new VBA is only carried over to the next epoch or considered for another base station if its marginal probability exceeds 0.5.

## 5. Experiments and Results

### 5.1. Experimental Setup

To validate the performance of the proposed method, we conducted experiments in the underground parking lot of Beijing University of Posts and Telecommunications, Beijing, China. We employed CDMA pseudo-orthogonal spreading code signals, similar to GPS signals, as the ranging signals. The signal configuration is detailed in Table 2. The signals were transmitted by time-synchronized positioning sources (developed based on the ZYNQ7020 platform) (Xilinx, San Jose, CA, USA) in a configuration that uses a common baseband module with a splitter connecting to multiple RF antennas. Simultaneously, we used a USRP B210 software-defined radio (SDR) to receive and collect the signals. We use a rod-type omnidirectional antenna with a frequency range of 3400–3600 MHz and a gain of 3 dBi. The LiDAR point clouds were acquired by a Helios 16 model LiDAR (RoboSense Technology Co., Ltd., Shenzhen, China). Both the LiDAR and the signal collector were co-located on a mobile terminal platform based on an unmanned ground vehicle. The solution process was executed on a computer with an Intel Core i5-12400 CPU (@2.5 GHz) (Intel, Santa Clara, CA, USA) and 16 GB RAM, running Ubuntu 20.04.6. The solution result output frequency was 1 Hz.

During the neural network training process, we used the Adam optimizer with its default parameters. The initial learning rate was set to 0.01, decaying by 5% after each epoch. The number of nearest neighbors *K* was set to 16. The scale of a single point cloud frame was approximately $10^{6}$ points. Both the training and inference processes were conducted on an NVIDIA RTX 4060 Ti GPU (Santa Clara, CA, USA).

**Figure 4:**
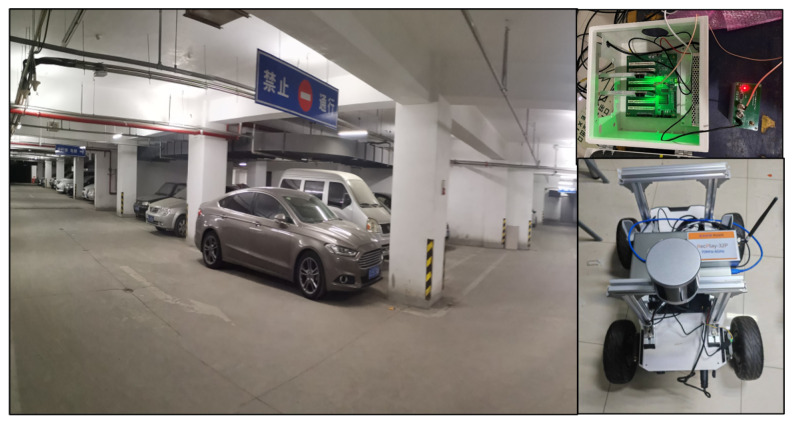
The actual test scenario, with the base station in the upper right and unmanned vehicle in the lower right.

The actual test scenario in the underground parking lot of Beijing University of Posts and Telecommunications is shown in Figure 4. The left side shows a photograph of the site. The right side shows a diagram of the base station layout and the unmanned vehicle equipped with LiDAR and signal collectors. The signals generated by the base station were connected via cables to different anchor points. The connecting cables and antenna anchors are visible in the photograph.

**Figure 5:**
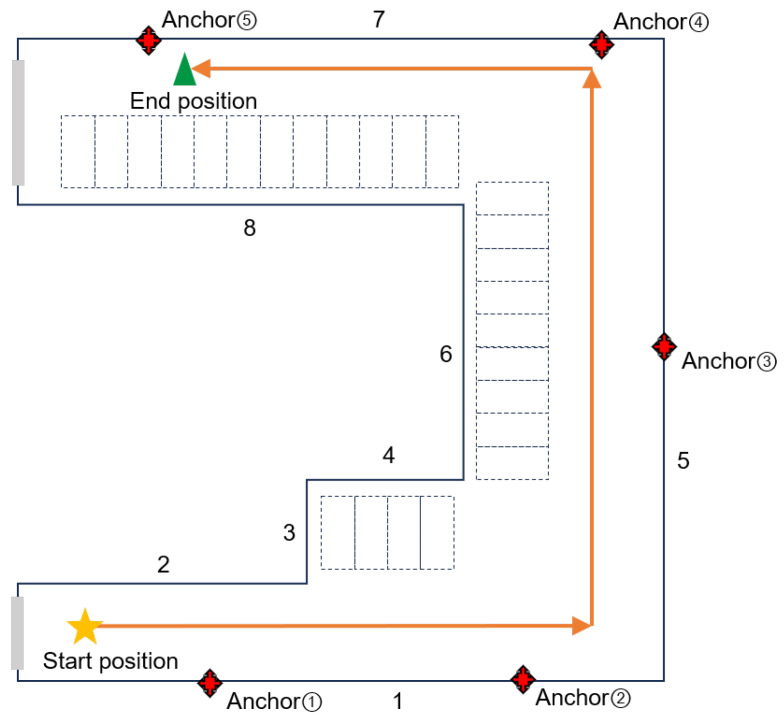
Anchor deployment and mobile terminal travel path.

The floor plan of the experimental path is shown in Figure 5. The area contains a total of 8 walls that cause signal reflections. The gray area represents a corridor connecting the entrance and exit. The red dots indicate the positions of the deployed anchors; we installed 5 physical anchors in total. The orange line shows the trajectory of the mobile terminal. As the terminal moves, signals from different anchors are subject to complex conditions such as occlusion and multipath effects. The reflective surface perception process was performed by pre-collecting point clouds with the LiDAR and completing the training beforehand. During the test, the vehicle traveled at a constant speed. The average speeds of the three route segments were 0.8 m/s, 1.1 m/s, and 0.7 m/s respectively, with no sudden acceleration or deceleration. In extreme cases where data transmission gets stuck, the unmanned vehicle is equipped with a preconfigured “timeout parking” mechanism (it automatically stops if the delay exceeds 2 s), but this mechanism was not triggered in this test.

### 5.2. Terminal Positioning Experiment

#### 5.2.1. Terminal Trajectory and Position Error

In this experiment, we compared the 2D positioning results of different methods. The Ground Truth is the calibrated true trajectory. Single Path represents the position obtained by conventional ranging observation solution, where the ranging observation is determined by the peak of the code correlation. It represents the basic positioning capability of the traditional method (without multipath processing), serving as a reference for the performance lower bound. Single Path FGO is the position optimized by factor graph based on the Single Path method. It is used to verify the effect of the factor graph acting alone. Multipath Compensation is the position calculated after multipath error correction based on environmental map compensation. It is a well-performing method in recent years, which uses prior information to simulate multipath signals. Multipath FGO is the proposed method in this paper, which is a multipath estimation approach based on semantic-associated factor graphs.

**Figure 6:**
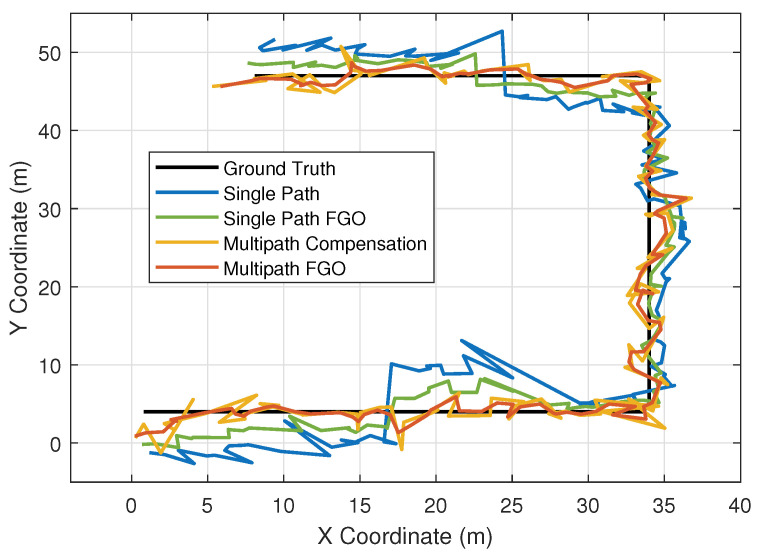
The trajectory of the terminal on the plane.

It can be observed that in Figure 6, due to adverse environmental effects such as anchor occlusion or multipath, the Single Path method exhibits significant errors in both the lower and upper areas of the figure. The Single Path FGO method, which uses factor graph optimization, has a smoothing effect that reduces these errors. In comparison, the Multipath Compensation method performs better than mere smoothing because it utilizes a prior environmental map model to simulate potential multipath signals in the code correlation peak, thereby compensating for errors. Our proposed Multipath FGO method also addresses multipath, but it further utilizes multipath information to increase the number of virtual anchors, resulting in greater stability.

**Figure 7:**
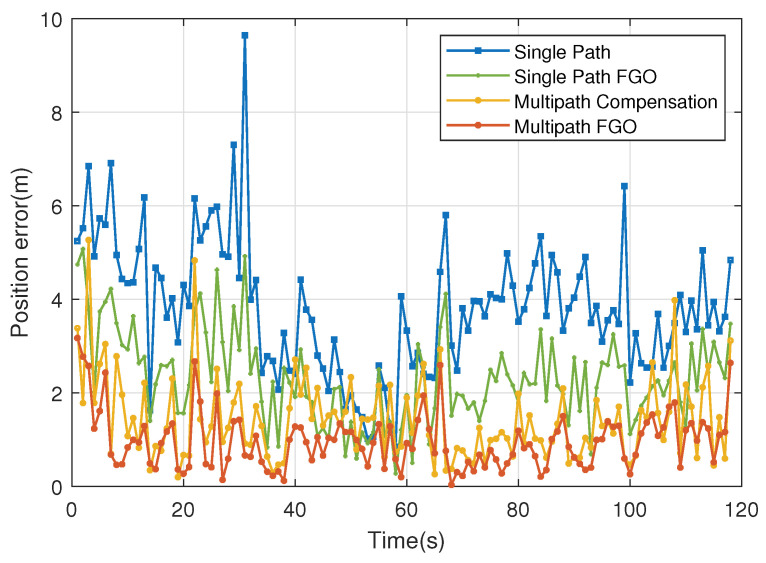
Two-dimensional positioning error.

We statistically analyzed the position error for each epoch, as shown in Figure 7. It can be seen that before 35 s, the Single Path error is large, indicating that the ranging observations are significantly affected by multipath and NLOS conditions, corresponding to the lower area of the terminal trajectory where measurements from Anchor 4 and Anchor 5 exhibit substantial deviations. Between 35 s and 65 s, the Single Path error is relatively small, as this segment has more Line-of-Sight observations and is less affected by multipath, corresponding to the right-side area of the trajectory. After 65s, the Single Path error increases again, similar to the pre-35 s period, where measurements from Anchor 1 and Anchor 2 show significant deviations. In areas with good observation conditions, the errors of all methods can be maintained around 2 m; however, in areas with poor observation conditions, methods that do not consider multipath exhibit more pronounced errors. Table 3 summarizes the error levels of all methods. Compared to the original positioning results, all metrics of Single Path FGO show improvement. For methods considering multipath processing, both the Multipath Compensation method and our proposed Multipath FGO method achieve RMSE within 2 m. Moreover, the Multipath FGO method delivers the best performance, improving accuracy by 32.1% compared to the multipath compensation approach.

#### 5.2.2. Comparison of Multiple Association Processes

To validate the impact of multi-epoch VBA states, measurements, and LiDAR perception triple constraints on position estimation, we compared the error distributions under four association scenarios. Association 1 represents estimation without inter-epoch association, which does not consider state transitions between epochs. Association 2 involves multi-epoch terminal state estimation without considering VBA states. Association 3 incorporates multi-epoch association between VBA states and measurements, but without LiDAR perception constraints on VBA states. Association 4 represents the full multi-epoch association between VBA states, measurements, and LiDAR perception.

**Figure 8:**
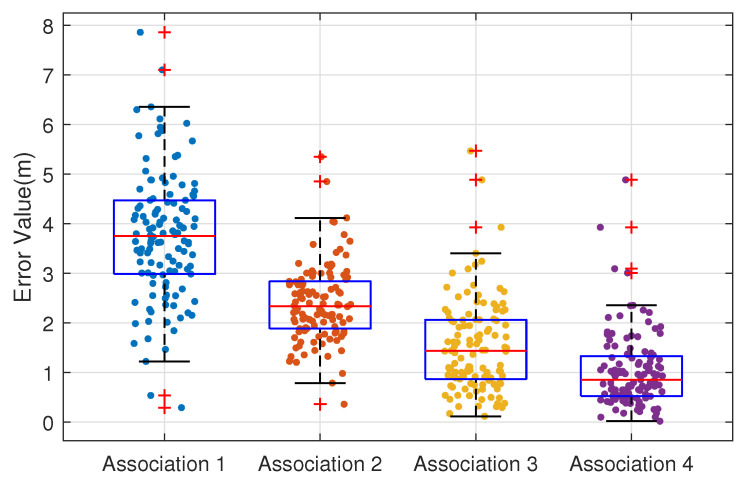
Error box plot under different data association methods.

The experimental error box plot is shown in Figure 8. It can be observed that Association 1 essentially performs discrete epoch-by-epoch estimation. Since it does not utilize state information across multiple epochs, it achieves the lowest accuracy with an average error of 3.74 m. By considering terminal state transitions across multiple epochs, Association 2 improves the accuracy to 2.38 m with a more concentrated distribution, demonstrating the effectiveness of multi-epoch information constraints. After incorporating virtual anchor state considerations, Association 3 utilizes every potential multipath component to update the terminal state, reducing the average error to 1.53 m. Finally, Association 4 adds LiDAR perception association, further constraining VBA states and improving terminal positioning accuracy to 1.03 m.

#### 5.2.3. Algorithm Speed Test

Table 4 presents the average single computation time of each method. The baseline method Single Path has the shortest computation time (1.3 ms), as it does not incorporate additional optimization modules. After integrating factor graph optimization, Single Path FGO increases the computational complexity, leading to a computation time of 10.9 ms. Multipath Compensation, which adds a multipath compensation module and requires ray tracing processing, has a computation time of 8.7 ms; meanwhile, the proposed Multipath FGO in this paper features a more complex computation process—since it integrates modules like point cloud neural network-based reflective surface detection and factor graph optimization—resulting in an average single computation time of 45.1 ms.

### 5.3. Multipath Estimation Experiment

#### 5.3.1. Virtual Anchor Position Estimation

**Figure 9:**
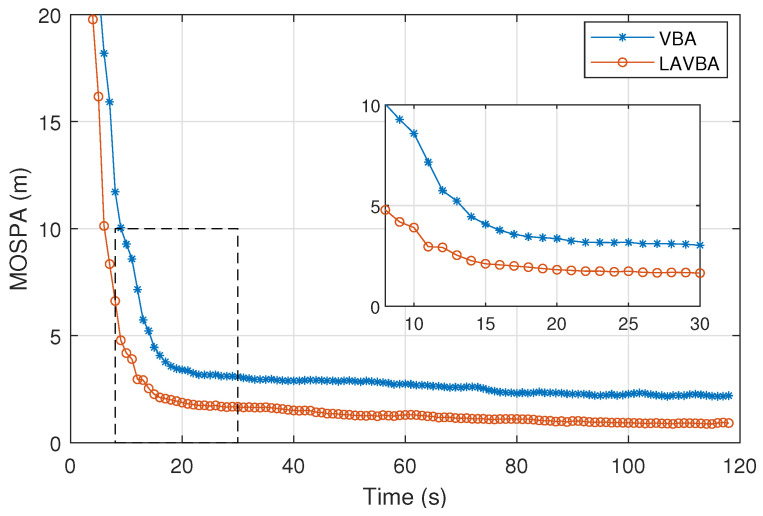
Virtual Anchor Position Estimation MOSPA.

To evaluate the accuracy of multipath information in our method, we estimated the position information of virtual anchors corresponding to each path. We first transformed the VBA state information into virtual anchor state information. We used the Optimal Subpattern Assignment (OSPA) metric to describe the virtual anchor position estimation error, which can measure the accuracy of multi-target state estimation [31]. Specifically, we used the Euclidean distance to construct a cost matrix between the estimated virtual anchor positions and the true virtual anchor positions, and found the optimal assignment scheme that minimizes the average distance sum between all matched target pairs. We statistically analyzed the impact of LiDAR perception assistance on virtual anchor estimation, as shown in Figure 9. The method without LiDAR assistance is labeled as VBA, while with LiDAR assistance is labeled as LAVBA.

It can be observed that when using only signal measurements to estimate VBA states, the error converges to 10 m after 8 s, to 5 m after 14 s, and finally stabilizes at 2.24 m. When using LiDAR-perceived reflective surface positions to assist VBA estimation, the error converges to 5 m after 8 s and finally stabilizes at 0.92 m. The LiDAR perception process accelerates the convergence of VBA state estimation and reduces the estimation error after convergence. This is because LiDAR directly outputs reflective surface positions, which can jointly constrain VBA states along with signal measurements.

#### 5.3.2. Reflective Surface Perception

To comprehensively evaluate the effectiveness of the proposed method, we compared PointCNN, PointNet, PointNet+CNN, and our proposed model on the same dataset. As shown in Table 5 and Table 6, experimental results were quantitatively compared regarding two dimensions: model complexity (parameter count, computational load) and regression accuracy (recognition precision, center point error, normal vector error, virtual anchor error). The recognition precision is calculated based on the reflective surface detection probability $p_{\mathrm{refl},k}$ after Hungarian matching, and a reflective surface is only considered to exist when $p_{\mathrm{refl},k}>0.5$. The virtual anchor error of the model output is calculated as $p_{\mathrm{mva}}=2n_{\mathrm{refl}}^{T}P_{l}^{T}n_{\mathrm{refl}}$. Analysis shows that our proposed model achieves optimal performance in potential reflective surface detection. Specifically, the model proposed in this paper achieves the highest recognition accuracy. In terms of the center point error (0.26 m) and normal vector error (0.76°), our model significantly outperforms other compared models, directly demonstrating its accuracy in spatial position and orientation estimation tasks. The computed virtual anchor error (1.82 m), as a comprehensive metric, further verifies the overall superiority of the model. In terms of the model complexity, our model’s parameter count (3.2 MB) is comparable to PointNet, but its computational complexity (17.8 GFLOPs) is higher than PointNet+CNN (13.4 GFLOPs). This indicates that the performance improvement does not stem from simple parameter stacking but from more efficient network architecture design. The introduced local feature aggregation and context-aware modules, although increasing some computational overhead, significantly enhance the understanding of point cloud geometric features, thereby achieving a breakthrough in accuracy. Our proposed method achieves significant performance improvement at an acceptable computational cost, enabling more accurate detection of potential reflective surface positions.

## 6. Conclusions and Future Work

This paper addresses the challenge of positioning accuracy degradation caused by multipath effects in complex indoor environments by proposing a LiDAR-assisted multipath estimation and localization method. The core innovation lies in constructing a tightly coupled perception-localization framework that significantly enhances positioning performance by fusing geometric information from LiDAR perception with wireless signal measurements. The key contributions include: (1) designing a deep learning-based reflective surface detection model for accurately extracting geometric features of potential reflectors from LiDAR point clouds; (2) establishing a unified factor graph optimization model that jointly estimates terminal states, Virtual Anchor (VA) states, wireless signal measurements, and LiDAR-perceived reflective surface information; and (3) introducing temporal state transition and multi-dimensional data association mechanisms to dynamically resolve the matching between multipath signals and reflective surfaces. Experimental results in real indoor scenarios demonstrate that the proposed method reduces the positioning Root Mean Square Error (RMSE) to 1.14 m, representing a 32.1% improvement compared to traditional multipath compensation approaches, while significantly lowering the Virtual Anchor estimation error to 0.92 m. This confirms the method’s effectiveness in enhancing positioning accuracy and robustness in complex indoor environments.

Future research will focus on multi-modal sensor fusion strategies, incorporating data from vision, IMU, and other sensors to further improve the system’s practicality and generalization capability. Specifically, we will explore deep integration of visual-inertial information to enhance positioning continuity and reliability in scenarios where LiDAR perception is limited (e.g., texture-less areas or rapid motion). Furthermore, we will investigate adaptive mechanism optimization to enhance the system’s scalability and real-time performance in large-scale dynamic environments. This research provides a new technical pathway for high-precision indoor positioning, with broad application prospects in fields such as autonomous vehicle indoor parking and smart warehouses. In the future, it can be adaptable to autonomous driving in GNSS-denied scenarios such as tunnels and underground parking lots, as well as position monitoring of unmanned equipment in industrial workshop scenarios.

## Figures and Tables

**Table 1:** Symbol Index Table.

Notation	Definition	Notation	Definition
$p_{\mathrm{pa}}^{\left(j\right)}$	2D position of physical anchor *j*	$p_{n}$	2D position of mobile terminal at time *n*
*j*	Index of physical anchor (1≤j≤J, *J* is total number of physical anchors)	$p_{\mathrm{va},s}^{\left(j\right)}$	Virtual anchor position of physical anchor *j* corresponding to reflective surface *s*
*n*	Index of time epoch (1≤n≤N, *N* is total number of epochs)	$p_{\mathrm{vba},s}$	Position of Virtual Base Anchor (VBA) corresponding to reflective surface *s*
*s*	Index of reflective surface/VBA (1≤s≤S, *S* is total number of reflective surfaces)	$p_{\mathrm{vba},k}$	LiDAR-perceived VBA position of *k*-th candidate reflective surface
*m*	Index of multipath component ($1\leq m\leq M_{n}^{\left(j\right)}$, $M_{n}^{\left(j\right)}$ is number of multipath components for anchor *j* at epoch *n*)	$x_{n}$	State vector of mobile terminal at epoch *n* ($x_{n}={\left[p_{n}^{T}\;v_{n}^{T}\right]}^{T}$, $v_{n}$ is velocity component)
*k*	Index of LiDAR-detected candidate reflective surface ($1\leq k\leq K_{n}$, $K_{n}$ is number of candidate surfaces at epoch *n*)	$r_{n,s}$	Binary existence variable of *s*-th reflective surface at epoch *n* (0: non-existent; 1: existent)
$z_{m,n}^{\left(j\right)}$	*m*-th multipath component measurement of anchor *j* at epoch *n*	λ	Retention probability of reflective surface across epochs (decay factor)
$v_{m,n}^{\left(j\right)}$	Zero-mean Gaussian measurement noise of $z_{m,n}^{\left(j\right)}$	$P_{\mathrm{th}}$	Pruning threshold for VBAs (pruned if retention probability < $P_{\mathrm{th}}$)

**Table 2:** Ranging Signal Configuration.

**Signal Center Frequency**	3.5 GHz
**Modulation Method**	BPSK
**Multiple Access Method**	CDMA
**Spreading Code Generation Method**	Weil Code Set
**Pseudo-code Length**	10,230
**Pseudo-code Frequency**	10.23 MHz

**Table 3:** Two-dimensional Positioning Error Statistics.

	Single Path	Single Path FGO	Multipath Compensation	Multipath FGO
RMSE(m)	4.07	2.51	1.68	1.14
Mean (m)	3.81	2.31	1.43	0.97
STD (m)	1.41	0.97	0.89	0.61
Max (m)	9.64	5.07	5.26	3.17
Min (m)	0.84	0.27	0.19	0.03

**Table 4:** Algorithm Running Time.

	Single Path	Single Path FGO	Multipath Compensation	Multipath FGO
Average Single Computation Time (ms)	1.3	10.9	8.7	45.1

**Table 5:** Network Model Performance.

	Recognition Precision	Center Point Error (m)	Normal Vector Error (°)	VBA Error (m)
PointCNN [14]	87.1%	0.34	1.27	2.37
PointNet [15]	83.7%	0.39	1.12	2.21
PointNet+CNN	88.5%	0.32	0.91	2.02
Proposed	91.2%	0.26	0.76	1.82

**Table 6:** Network Model Complexity.

	Network Parameters (MB)	Computational Complexity (G FLOPs)
PointCNN	0.6	25.3
PointNet	3.2	14.7
PointNet+CNN	2.6	13.4
Proposed	3.2	17.8

## Data Availability

The original contributions presented in the study are included in the article, further inquiries can be directed to the corresponding authors.
